# Promoting endogenous mutual-aid older adults care in rural areas for healthy aging: an evolutionary game-based analytical framework

**DOI:** 10.3389/fpubh.2026.1790640

**Published:** 2026-03-12

**Authors:** Jian Wu, Naiyuan Fu, Jing Yang, Jing Gu

**Affiliations:** 1 Laboratory for Digital Intelligence & Health Governance, Nanjing Medical University, Nanjing, China; 2 School of Health Policy and Management, Nanjing Medical University, Nanjing, Jiangsu, China; 3 School of Humanities and Management, Ningxia Medical University, Yinchuan, Ningxia, China; 4 Department of Organization and Human Resources, The First Affiliated Hospital of Nanjing Medical University, Nanjing, China

**Keywords:** rural endogenous mutual-aid older adults care model, healthy aging, evolutionary game theory, micro-level mechanisms, MATLAB simulation

## Abstract

**Introduction:**

Against the backdrop of rapid population aging and widening health vulnerabilities among older adults in rural China, the erosion of traditional family-based care has posed significant challenges to achieving healthy aging and equitable older adults care. Promoting an endogenous mutual-aid older adults care model has therefore become a critical issue in public health and governance.

**Methods:**

This paper utilizes an evolutionary game model to analyze dynamic interactions among local governments, social organizations, and the rural older adults.

**Results:**

MATLAB simulations identify four critical drivers: willingness to participate, reward intensity, punishment severity, and reputation loss. Results reveal a paradox where high willingness from social actors accelerates local governments' tendency toward negative support. This paradox arises because the proactive engagement of social organizations and older adults may alleviate perceived pressure on local governments, thereby diminishing their motivation to provide active support. However, stricter penalties imposed by higher-level governments consistently promote positive choices across all parties. Furthermore, while reputation risks motivate government support, they may simultaneously deter older adults participation.

**Discussion:**

This study proposes strategic recommendations: implementing stricter penalties for government non-compliance, optimizing incentives for social organizations, and offering targeted guidance to the rural older adults to foster their active engagement in mutual care activities. These measures aim to facilitate the sustainable development of an effective rural mutual aid care ecosystem.

## 1 Introduction

Driven by both the national strategy of “healthy aging” and the pressing reality of rural areas facing aging before affluence and weakening family-based eldercare functions (1), the traditional role of family-based eldercare has gradually diminished, making rural eldercare issues increasingly prominent (2). This situation necessitates urgent exploration and establishment of older adults care service models specifically tailored to rural contexts (3). Against this backdrop, the State Council of China introduced a significant policy initiative in its 2018 government work report, formally proposing the development of mutual older adults care systems. This direction was further reinforced by the 20th Central Committee of the Communist Party of China, which emphasized the integration of mutual older adults care with medical services to achieve positive population aging outcomes. Consequently, exploring the establishment of a new model of mutual care for the older adults has become one of the important contents of solving the rural pension problem at this stage. Mutual support for the older adults can be divided into exogenous and endogenous modes according to the dominant subject (3). The exogenous mutual care model, implemented through top-down governmental directives or market-driven entities, has encountered several operational challenges, including low service acceptance rates, suboptimal assistance outcomes, and limited sustainability (4). In contrast, the endogenous mutual care model relies on the internal strength and resource integration of villages, and initiates the practice of mutual care from the bottom up (5), which can effectively solve the problem of insufficient power for sustainable development under the exogenous mutual care model (26). Therefore, exploring the promotion mechanism of rural endogenous mutual care model has become an important direction to solve the problem of sustainable development of rural mutual care.

The efficacy of the older adults care model is significantly compromised by the ambiguous delineation of interests and responsibilities among participating entities (6), which restricts the effective construction and development of the endogenous mutual care model in rural areas. According to neo-endogenous development theory, endogenous mutual care for the older adults can achieve sustainable development through the effective interaction of internal and external forces, which is actively guided and supported by external forces such as local governments and social organizations, and drives the development of internal forces in the countryside (7). The local government stimulates the enthusiasm and creativity within the villages through policy support, financial investment and technical guidance; while social organizations provide support such as training, volunteer services and resource integration to promote the establishment and improvement of mutual aid older adults care mechanisms within the rural areas (8, 9). As the core participant of rural internal forces and mutual care (3), the participation behavior of the older adults directly affects the sustainable development of rural mutual care (10). Therefore, the effective interaction between local governments, social organizations and rural older adults people is of great significance in promoting the sustainable development of endogenous mutual care in rural areas.

In recent years, the application of multi-subject evolutionary game as an analytical framework for the governance of conflict of interest issues in complex systems in the field of senior care has opened up new paths for the study of the development of older adults care institutions. For example, the sustainable development of smart older adults service platforms (11). Using evolutionary game modeling to explore how healthcare and social care can work together to serve older people in China (12). Considering the intelligent transformation path and development incentive mechanism of China's intelligent community older adults industry under market dominance from the perspective of multi-subject gaming (13). Utilizing a power-interest matrix develop the stakeholder collaboration mechanism, to promote older adults community retrofit projects in China (14). A study has been conducted to present the effect of government subsidies on the price, quality and quantity of demand for older adults services under different subsidy policies by developing a Nash game model consisting of older adults customers and two older adults service providers with different levels of infrastructure (15). Solving the problem of regulating the service quality of pension PPP projects in China by constructing an evolutionary game model between private investors and government regulators (16). In summary, it can be seen that the existing research has made useful exploration of the cooperation mechanism between the government and senior care institutions, which provides a good reference for the formation of ideas in this paper. Based on Li's tripartite model (17), we established a multi-stakeholder evolutionary game model that focuses on constructing an endogenous mutual older adults care framework comprising three core components: endogenous forces as the foundation, governmental forces as the supporting mechanism, and social organization forces as the synergistic element. Through this framework, the study analyzes the internal mechanisms driving behavioral interactions among participants in rural endogenous mutual older adults care systems.

The contributions of this paper are summarized as follows. Firstly, constructing a game model of the evolution of rural endogenous mutual care model from the perspective of multi-subject game with the participation of local government, social organizations and rural older adults. Secondly, this paper regards the rural older adults as the participants in the process of mutual care, emphasizes their subjective status and mobility in mutual care, and reveals how the local government, social organizations, and the rural older adults can effectively interact with each other as participants in mutual care to promote the construction and development of endogenous rural mutual care.

The remainder of the paper is organized as follows: The hypothesis and the mathematical model to formulate the behavioral strategies of the participants are presented in Section 2 and Section 3. The evolutionary stability analysis in Section 4 is validated, demonstrating that the different elements of the replication dynamics system significantly influence both the process and outcomes of the evolutionary game. Finally, section 5 summarizes the conclusions of the study, and makes suggestions for management and government.

## 2 Model assumption and description

### 2.1 Description of the problem

**Figure 1:**
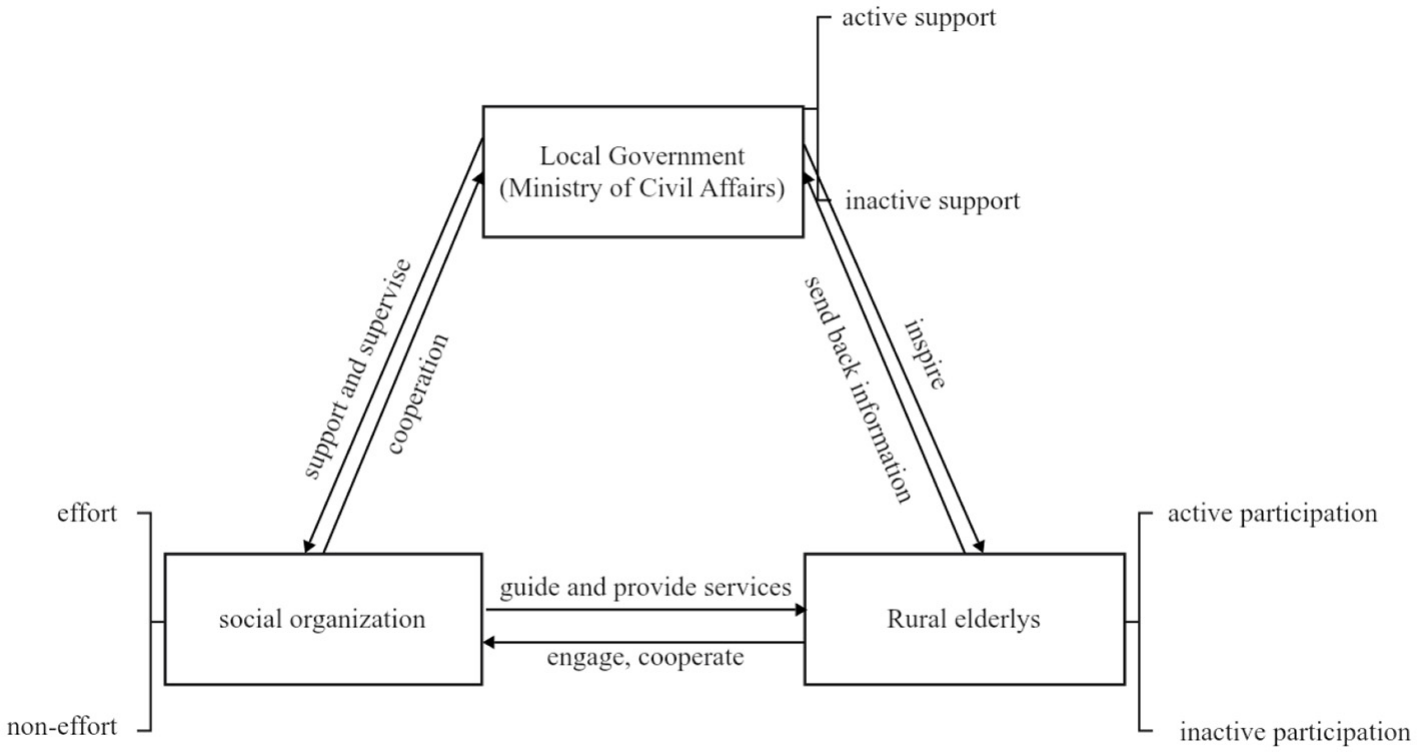
Interaction relationship between the three parties involved in the game behavior.

From the endogenous perspective to explore the generation of rural endogenous mutual-help older adults care model under the multi-subjects, mainly including the local government, social organizations and rural older adults, the game relationship between the three and the behavioral choice strategy is shown in Figure 1.

In the endogenous rural mutual care model, the local government acts as a guide, plays the role of a service-oriented and responsible government, and provides financial and policy support and supervision to social organizations in the form of subsidies or purchased services (18), while at the same time incentivizing the older adults to actively participate in mutual care, creating conditions for promoting rural mutual care, and granting a certain degree of autonomy to the rural areas and social organizations. Social organizations, as promoters and collaborative subjects of mutual care for the older adults, enhance the self-sufficiency and reciprocal assistance capabilities of the rural older adults demographic through the systematic absorption and integration of public welfare resources and corporate philanthropic capital (19, 20). Within this mutual care, the older adults are both service providers and recipients, and through self-care and mutual help, they assume certain care responsibilities, forming the core strength of mutual care (21).

### 2.2 Model assumption and parameter descriptions

Based on the problem description and the relationships among the three parties' game behaviors, this study establishes the following assumptions to construct a three-party evolutionary game model for the rural endogenous mutual care system:

#### 2.2.1 Assumption 1: the assumption of limited rationality of participating subjects

Within the rural endogenous mutual care model, the three participating subjects gradually adjust their strategies to maximize their own interests. Their decisions are influenced by their learning ability, game outcomes, and the behavioral choices of other subjects. Therefore, it is assumed that all agents are boundedly rational and adjust their strategies through imitation. Additionally, the information among the subjects is not entirely asymmetric, and their interactions are influenced by game randomness and behavioral dynamics.

#### 2.2.2 Assumption 2: behavioral strategy set assumption of participating subjects

The set of strategies of the local government is {active support, inactive support} with probabilities *x*, 1 − *x*. “Positive support” includes policy and financial support for social organizations. It also involves measures to promote and encourage older adults participation in mutual care activities. As well as to enhance the reputation and prestige of the social organizations, etc. Conversely, “Negative support” refers to the government's lack of active give any support to social organizations, as well as indicates the absence of efforts to promote and encourage older adults participation in mutual care activities.

The strategy set for social organizations is {effort, non-effort}, with probabilities *y*, 1 − *y*. The “effort” strategy involves actively mobilizing social resources to provide high-quality services to the rural older adults. It also includes establishing localized self-operating mutual aid service systems and fostering village-based operation teams (20). Conversely, the “non-effort” strategy refers to providing substandard services and lacking initiative in developing self-sustaining mutual aid systems within rural communities.

The set of strategies for the older adults is {active participation, inactive participation} with probabilities *z*, 1 − *z*. “Active participation” is older adults individuals' direct engagement in mutual care service provision, coupled with their collaborative involvement in social organizations' management and operational arrangements. In contrast, “Inactive participation” is defined by either minimal engagement or complete non-participation of older adults individuals in mutual care activities. *x, y, z* ∈ [0, 1]. The willingness to participate here specifically refers to the rural older adults's intrinsic motivation to engage in mutual-aid activities, which is influenced by factors such as perceived benefits, health conditions, and social connectedness.

#### 2.2.3 Assumption 3: relevant parameter assumptions and definitions

The gains and losses of local governments are analyzed as follows. When providing active support, the government incurs costs through investments in human, material, and financial resources, denoted as *C*_1_. The corresponding benefits, represented as *R*_1_, include improvements in the government's public image and enhancements in social welfare. The cost of negative support is denoted as *C*_2_(*C*_2_ < *C*_1_). Reputation loss of local goverment, denoted as *D*_1_, refers to the damage to credibility and performance evaluation that local governments incur when they fail to actively support mutual care initiatives. Additionally, punitive measures from higher levels of governmental echelons, including administrative sanctions and financial penalties, are represented by *P*_1_. When the government assumes a passive stance, the participation of social organizations generates supplementary benefits, quantified as *R*_2_. Furthermore, active older adults participation reduces governmental burden, yielding additional benefits denoted as *R*_3_. The social organizations' lack of effort resulting in losses such as damage to the Government's image and waste of resources is recorded as *D*_2_. Losses to the Government resulting from the lack of active participation of older persons, which leads to poor policy results in mutual-help projects for the older adults and ineffective utilization of resources, are recorded as *D*_3_.

The gains and losses of social organizations are analyzed as follows. The cost of the social organization's efforts to participate is denoted as *C*_3_, the benefit is denoted as *R*_4_. When local governments provide active support, social organizations receive benefits, including governmental recognition and monetary incentives, represented as *R*_*so*_. When the local government chooses inactive support, the additional benefit gained by social organizations through the effort strategy is recorded as *R*_6_. In this case, the wastage of resources such as human, material and financial resources as well as the loss of reputation of the social organization as a result of the older adults choosing the strategy of inactive participation is recorded as *D*_5_. The cost incurred by social organizations when choosing a non-effort strategy is recorded as *C*_4_(*C*_4_ < *C*_3_), The self trust crisis and reputation damage caused at the same time are recorded as *D*_4_, and the administrative and economic penalties imposed by the government are recorded as *P*_*so*_. When social organizations do not make efforts, the active participation of the older adults brings additional performance and positive social benefits to the social organizations, denoted as *R*_5_ (22), denoted as *D*_6_.

The investment of time and human capital by older adults individuals in community participation is denoted as *C*_5_, while the corresponding benefits, including enhanced quality of life and reduced older adults care expenses, are represented as *R*_7_. In scenarios with active government support, participating older adults individuals receive both material and spiritual incentives, designated, which are recorded as *R*_*e*_. Furthermore, under the government actively supports, older adults individuals who opt inactive participation still derive benefits from public infrastructure investments made by local authorities, which is denoted as *R*_8_.

The main notations of the model and their definitions are listed in Table 1.

**Table 1:** Variables and definitions.

**Variables**	**Meaning of variables**
*C*_1_	Costs invested in active support by local governments
*C*_2_	Costs invested in negative support by local governments (*C*_2_ < *C*_1_)
*P*_1_	Penalties imposed by higher-level governments on local governments when they provide negative support
*R*_1_	The benefits obtained from the active support of local governments
*R*_2_	Additional benefits to the government when local governments are negatively supportive and social organizations choose to make an effort to participate
*R*_3_	Additional benefits to government when local governments are negatively supportive and older people choose to be actively involved
*D*_1_	Loss of government credibility, performance, etc., when local governments are negatively supportive
*D*_2_	Damages to the Government caused by the non-effort of social organizations to participate when the local government is actively supporting them
*D*_3_	Losses to the government from the inactive participation of older people when local governments are actively supporting them
*C*_3_	The cost of investment in social organization's choice of effort strategy
*C*_4_	The cost invested by social organizations when choosing non-effort (*C*_4_ < *C*_3_)
*P*_*so*_	Penalties for social organizations that do not make an effort to participate when local governments actively support them
*R*_*so*_	Awards received by social organizations for choosing effort strategies when actively supported by the government
*R*_4_	The benefits obtained from the efforts of social organizations to participate
*R*_5_	Additional benefits to social organizations from the active participation of older persons when social organizations do not make an effort
*R*_6_	Additional benefits to social organizations from their efforts to participate when government is negative support
*D*_4_	Crisis of self-confidence and loss of reputation caused by social organizations choosing a no-effort strategy
*D*_5_	Damage to social organizations caused by the inactive participation of older persons
*C*_5_	The cost of active participation by the older adults
*R*_*e*_	Rewards received by older adults people who actively participate with the government's active support
*R*_7_	Benefits derived from the active participation of the older adults
*R*_8_	The “hitchhiking” benefits of not actively participating by older persons when actively supported by local governments
*D*_6_	Damage to the older adults caused by the non-effort of social organizations to participate
*x*	Probability of positive support by local government
*y*	Probability of social organization efforts
*z*	Probability of positive involvement of the older adults

### 2.3 Establishment of the model

According to the aforementioned hypotheses, the payment matrix of social organization, local governments and the older adults in rural areas is constructed, which is shown in Table 2.

**Table 2:** Payment matrix for all players.

**Local governments**	**Social organization**	**The older adults**	
		**Participate actively** (*z*)	**Not actively participate** (1 − *z*)
Active support (*x*)	Effort (*y*)	*R*_1_ − *C*_1_ − *R*_*so*_ − *R*_*e*_	*R*_1_ − *C*_1_ − *R*_*so*_ − *D*_3_
		*R*_4_ + *R*_*so*_ − *C*_3_	*R*_4_ + *R*_*so*_ − *C*_3_ − *D*_5_
		*R*_7_ + *R*_*e*_ − *C*_5_	*R*_8_
	Non–effort (1 − *y*)	*R*_1_ + *P*_*so*_ − *C*_1_ − *R*_*e*_ − *D*_2_	*R*_1_ + *P*_*so*_ − *C*_1_ − *D*_2_ − *D*_3_
		*R*_5_ − *P*_*so*_ − *C*_4_ − *D*_4_	− *C*_4_ − *P*_*so*_ − *D*_4_ − *D*_5_
		*R*_7_ + *R*_*e*_ − *C*_5_ − *D*_6_	*R*_8_
Negative support (1 − *x*)	Effort (*y*)	*R*_2_ + *R*_3_ − *C*_2_ − *P*_1_ − *D*_1_	*R*_2_ − *C*_2_ − *P*_1_ − *D*_1_
		*R*_4_ + *R*_6_ − *C*_3_	*R*_4_ + *R*_6_ − *C*_3_ − *D*_5_
		*R*_7_ − *C*_5_	0
	Non–effort (1 − *y*)	*R*_3_ − *C*_2_ − *P*_1_ − *D*_1_	− *C*_2_ − *P*_1_ − *D*_1_
		− *C*_4_ + *R*_5_ − *D*_4_	− *C*_4_ − *D*_4_ − *D*_5_
		− *D*_6_	0

### 2.4 The construction of tripartite game revenue expectation function

According to the payment income matrix in Table 2, the expected income functions and equilibrium strategy points are constructed for local government, social organizations, and the older adults.

(1) The expected revenue function of local government

If the local government choose to implement positive support (negative support), the payoff function is *U*_1_(*U*_2_), and the average expected revenue of the local government is *U*_*g*_.


$$\begin{array}{l} U_{1}=z\left(D_{3}-R_{e}\right)+y\left(-R_{so}-P_{so}+D_{2}\right)+\\ \left(R_{1}+P_{so}-C_{1}-D_{2}-D_{3}\right)\\ U_{2}=zR_{3}+yR_{2}+\left(-C_{2}-P_{1}-D_{1}\right)\\ U_{g}=xU_{1}+\left(1-x\right)U_{2} \end{array}$$ (1)

(2) The expected revenue function of social organization

If the social organization choose to implement effort (non effort), the payoff function is *U*_3_(*U*_4_), and the average expected revenue of the social organization is *U*_*s*_.


$$\begin{array}{l} U_{3}=x\left(R_{so}-R_{6}\right)+\left(zD_{5}+R_{6}+R_{4}-C_{3}-D_{5}\right)\\ U_{4}=x\left(-P_{so}\right)+z\left(R_{5}+D_{5}\right)+\left(-C_{4}-D_{4}-D_{5}\right)\\ U_{s}=yU_{3}+\left(1-y\right)U_{4} \end{array}$$ (2)

(3) The expected revenue function of the older adults

If the older adults chooses to implement active participation (Not actively participate), the payoff function is *U*_5_(*U*_6_), and the average expected revenue of the older adults is *U*_*e*_.


$$\begin{array}{l} U_{5}=x\left[y\left(R_{7}-C_{5}\right)+\left(R_{7}+R_{e}-C_{5}\right)\right]+\\ y\left(R_{7}-C_{5}+D_{6}\right)-D_{6}\\ U_{6}=xR_{8}\\ U_{e}=zU_{5}+\left(1-z\right)U_{6} \end{array}$$ (3)

### 2.5 Evolutionary strategy solution based on replication dynamic equation

(1) The replicator dynamic equation of the local governments can be written:


$$\begin{array}{l} F(x)=\frac{dx}{dt}=x(U_{1}-U_{g})=x(1-x)(U_{1}-U_{2})\\ =x(1-x)[z(D_{3}-R_{e}-R_{3})+y(-R_{so}-P_{so}+D_{2}-R_{2})\\ \;+(R_{1}+P_{so}-C_{1}-D_{2}-D_{3}+C_{2}+P_{1}+D_{1})] \end{array}$$ (4)

*F*(*x*) = *dx*/*dt* = 0, the solution obtained may be the point of evolutionary stable process.

When $y=y_{0}=\frac{z\left(D_{3}-R_{e}-R_{3}\right)+\left(R_{1}+P_{so}-C_{1}-D_{2}-D_{3}+C_{2}+P_{1}+D_{1}\right)}{R_{so}+P_{so}+D_{2}+R_{2}}$, *F*(*x*) = 0, the system reaches an evolutionarily stable state. indicating that the proportion of local governments selecting either “positive support” or “negative support” strategies remains constant over time. According to the stability theorem of differential equations (23), the evolutionary stable strategy is required to satisfy *F*(*x*) = 0 and *dx*/*dt* < 0.

When $y\neq y_{0}=\frac{z\left(D_{3}-R_{e}-R_{3}\right)+\left(R_{1}+P_{so}-C_{1}-D_{2}-D_{3}+C_{2}+P_{1}+D_{1}\right)}{R_{so}+P_{so}+D_{2}+R_{2}}$, let *F*(*x*) = 0, then *x* = 0 and *x* = 1 are two stable points. Derivation of *F*(*x*):


$$\begin{array}{l} F^{\prime }(x)=(1-2x)[z(D_{3}-R_{e}-R_{3})+y(-R_{so}-P_{so}+D_{2}-R_{2})\\ +(R_{1}+P_{so}-C_{1}-D_{2}-D_{3}+C_{2}+P_{1}+D_{1})] \end{array}$$ (5)

In this case, the analysis can be divided into two scenarios:

1) When $\frac{z\left(D_{3}-R_{e}-R_{3}\right)+\left(R_{1}+P_{so}-C_{1}-D_{2}-D_{3}+C_{2}+P_{1}+D_{1}\right)}{R_{so}+P_{so}+D_{2}+R_{2}}<y<1$, the $F^{\prime }\left(x\right){|}_{x=0}>0$, $F^{\prime }\left(x\right){|}_{x=1}<0$, At this time, *x* = 1 is the evolutionary dynamic equilibrium point, that is, when the probability of social organization efforts is greater than $\frac{z\left(D_{3}-R_{e}-R_{3}\right)+\left(R_{1}+P_{so}-C_{1}-D_{2}-D_{3}+C_{2}+P_{1}+D_{1}\right)}{R_{so}+P_{so}+D_{2}+R_{2}}$, local governments choose to actively support.

2) When $0<y<\frac{z\left(D_{3}-R_{e}-R_{3}\right)+\left(R_{1}+P_{so}-C_{1}-D_{2}-D_{3}+C_{2}+P_{1}+D_{1}\right)}{R_{so}+P_{so}+D_{2}+R_{2}}$, $F^{\prime }\left(x\right){|}_{x=0}<0,$ $F^{\prime }\left(x\right){|}_{x=1}>0$, *x* = 0 is the evolutionary dynamic equilibrium point, that is, when the probability of effort from social organization is less than $\frac{z\left(D_{3}-R_{e}-R_{3}\right)+\left(R_{1}+P_{so}-C_{1}-D_{2}-D_{3}+C_{2}+P_{1}+D_{1}\right)}{R_{so}+P_{so}+D_{2}+R_{2}}$, local governments choose negative support.

(2) The replicator dynamic equation of the social organization can be formulated as follows:


$$\begin{array}{l} F(y)=\frac{dy}{dt}=y(U_{3}-U_{s})=y(1-y)(U_{3}-U_{4})\\ =y(1-y)[x(R_{so}+P_{so}-R_{6})-z(R_{5})+\\ \left(R_{6}+R_{4}-C_{3}+C_{4}+D_{4})\right] \end{array}$$ (6)

Let *F*(*y*) = *dy*/*dt* = 0, the solution obtained may be the point of evolutionary stable process.

When $x=x_{0}=\frac{z\left(R_{5}\right)-\left(R_{6}+R_{4}-C_{3}+C_{4}+D_{4}\right)}{R_{so}+P_{so}-R_{6}}$, *F*(*y*) = 0, This shows that all *y* are in an evolutionary stable state, that is, the proportion of social organizations choosing “effort” and “non- effort”, and this proportion will not change with time. According to the stability theorem of differential equations, the evolutionary stability strategy is required to satisfy *F*(*y*) = 0 and *dy*/*dt* < 0.

When $x\neq x_{0}=\frac{z\left(R_{5}\right)-\left(R_{6}+R_{4}-C_{3}+C_{4}+D_{4}\right)}{R_{so}+P_{so}-R_{6}}$, *F*(*y*) = 0 , Then *y* = 0 and *y* = 1 are two stable points. By derivation, we get:


$$\begin{array}{l} F^{\prime }(y)=(1-2y)[x(R_{so}+P_{so}-R_{6})-z(R_{5})+\\ \;(R_{6}+R_{4}-C_{3}+C_{4}+D_{4})] \end{array}$$ (7)

From Equation 7, we have two cases to discuss:

1) When $\frac{z\left(R_{5}\right)-\left(R_{6}+R_{4}-C_{3}+C_{4}+D_{4}\right)}{R_{so}+P_{so}-R_{6}}<x<1$, $F^{\prime }\left(y\right){|}_{y=0}>0,F^{\prime }\left(y\right){|}_{y=1}<0$, *y* = 1 represents the evolutionary dynamic equilibrium point. This occurs when the probability of active support from local governments is greater than $\frac{z\left(R_{5}\right)-\left(R_{6}+R_{4}-C_{3}+C_{4}+D_{4}\right)}{R_{so}+P_{so}-R_{6}}$. In this case, social organizations tend to choose the effort strategy.

2) When $0<x<\frac{z\left(R_{5}\right)-\left(R_{6}+R_{4}-C_{3}+C_{4}+D_{4}\right)}{R_{so}+P_{so}-R_{6}}$, $F^{\prime }\left(y\right){|}_{y=0}<0,F^{\prime }\left(y\right){|}_{y=1}>0$, *y* = 0 is the evolutionary dynamic equilibrium point, that is, when the probability of active support from local governments is less than $\frac{z\left(R_{5}\right)-\left(R_{6}+R_{4}-C_{3}+C_{4}+D_{4}\right)}{R_{so}+P_{so}-R_{6}}$, social organizations choosing “non-effort”.

(3) The replicator dynamic equation of the older adults can be formulated as follow:


$$\begin{array}{l} F(z)=\frac{dz}{dt}=z(U_{5}-U_{e})=z(1-z)(U_{5}-U_{6})\\ =z(1-z)[xy(-R_{7}+C_{5})+x(R_{7}+R_{e}-C_{5}-R_{8})\\ +y(R_{7}-C_{5}+D_{6})-D_{6}] \end{array}$$ (8)

*F*(*z*) = *dz*/*dt* = 0, the solution obtained may be the point of evolutionary stable process.

When $x=x_{0}=\frac{-y\left(R_{7}-C_{5}+D_{6}\right)+D_{6}}{y\left(C_{5}-R_{7}\right)+\left(R_{7}+R_{e}-C_{5}-R_{8}\right)}$, *F*(*z*) = 0, This shows that all the Z values are in an evolutionary stable state, that is, the proportion of the older adults choosing “active participation” and “inactive participation” will not change with time. According to the stability theorem of differential equations, the evolutionary stability strategy is required to satisfy *F*(*z*) = 0 and *dz*/*dt* < 0.

When $x\neq x_{0}=\frac{-y\left(R_{7}-C_{5}+D_{6}\right)+D_{6}}{y\left(C_{5}-R_{7}\right)+\left(R_{7}+R_{e}-C_{5}-R_{8}\right)}$, let *F*(*z*) = 0, *z* = 0 and *z* = 1 are two stable points. By derivation, we get Equation 9


$$\begin{array}{l} F^{\prime }(z)=(1-2z)[xy(-R_{7}+C_{5})+x(R_{7}+R_{e}-C_{5}-R_{8})\\ \;+y(R_{7}-C_{5}+D_{6})-D_{6}] \end{array}$$ (9)

Discussion in two cases:

1) When $\frac{-y\left(R_{7}-C_{5}+D_{6}\right)+D_{6}}{y\left(C_{5}-R_{7}\right)+\left(R_{7}+R_{e}-C_{5}-R_{8}\right)}$<*x* < 1, $F^{\prime }\left(z\right){|}_{z=0}>0,F^{\prime }\left(z\right){|}_{z=1}<0$, *z* = 1 is the evolutionary dynamic equilibrium point, that is, when the probability of active support from local governments is greater than $\frac{-y\left(R_{7}-C_{5}+D_{6}\right)+D_{6}}{y\left(C_{5}-R_{7}\right)+\left(R_{7}+R_{e}-C_{5}-R_{8}\right)}$, the older adults chooses to participate actively.

2) When $0<x<\frac{-y\left(R_{7}-C_{5}+D_{6}\right)+D_{6}}{y\left(C_{5}-R_{7}\right)+\left(R_{7}+R_{e}-C_{5}-R_{8}\right)}$, $F^{\prime }\left(z\right){|}_{z=0}<0,F^{\prime }\left(z\right){|}_{z=1}>0$, *z* = 0 is the evolutionary dynamic equilibrium point, that is, when the probability of effort from social organization is less than $\frac{-y\left(R_{7}-C_{5}+D_{6}\right)+D_{6}}{y\left(C_{5}-R_{7}\right)+\left(R_{7}+R_{e}-C_{5}-R_{8}\right)}$, the older adults chooses to participate actively.

## 3 Model analysis

### 3.1 Model stability

By setting *F*(*x*) = 0, *F*(*y*) = 0, *F*(*z*) = 0, the equilibrium point of the dynamic process of evolutionary game are determined. In the asymmetric games, if the equilibrium of the evolutionary game an evolutionarily stable strategy, the game must be a strict Nash equilibrium. Since a strict Nash equilibrium is also a pure strategy equilibrium, so the dynamic mixed strategy equilibrium of the asymmetric game must not be an evolutionary stable equilibrium (24). Therefore, for the evolutionary game of tripartite participation in the rural endogenous mutual pension model, only the pure strategy, that is, the evolutionary stability of the points *E*_1_ = (0, 0, 0), *E*_2_ = (0, 0, 1), *E*_3_ = (0, 1, 0), *E*_4_ = (1, 0, 0), *E*_5_ = (0, 1, 1), *E*_6_ = (1, 1, 0), *E*_7_ = (1, 0, 1), *E*_8_ = (1, 1, 1) are considered. To evaluate the stability properties of these equilibrium points, we derive the Jacobian matrix by computing the first-order partial derivatives with respect to the relevant variables, as expressed in Equation 10. The results are shown in Table 3.


$$\begin{array}{l} J=\left(\begin{array}{ccc} b_{11} & b_{12} & b_{13}\\ b_{21} & b_{22} & b_{23}\\ b_{31} & b_{32} & b_{33} \end{array}\right) \end{array}$$ (10)

$$\begin{array}{l} b_{11}=\frac{\partial F(x)}{\partial x}=(1-2x)[z(D_{3}-R_{e}-R_{3})+y(-R_{so}-P_{so}\\ +D_{2}-R_{2})+(R_{1}+P_{so}-C_{1}-D_{2}-D_{3}+C_{2}+P_{1}+D_{1})] \end{array}$$ (11)

$$\begin{array}{l} b_{12}=\frac{\partial F\left(x\right)}{\partial y}=x\left(1-x\right)\left({-R}_{so}-P_{so}-D_{2}-R_{2}\right) \end{array}$$ (12)

$$\begin{array}{l} b_{13}=\frac{\partial F\left(x\right)}{\partial z}=x\left(1-x\right)\left(D_{3}-R_{e}-R_{3}\right) \end{array}$$ (13)

$$\begin{array}{l} b_{21}=\frac{\partial F\left(y\right)}{\partial x}=y\left(1-y\right)\left(R_{so}+P_{so}-R_{6}\right) \end{array}$$ (14)

$$\begin{array}{l} b_{22}=\frac{\partial F(y)}{\partial y}=(1-2y)[x(R_{so}+P_{so}-R_{6})-z(R_{5})\\ \;+(R_{6}+R_{4}-C_{3}+C_{4}+D_{4})] \end{array}$$ (15)

$$\begin{array}{l} b_{23}=\frac{\partial F\left(y\right)}{\partial z}=y\left(1-y\right)\left(-R_{5}\right) \end{array}$$ (16)

$$\begin{array}{l} b_{31}=\frac{\partial F(z)}{\partial x}=z(1-z)[y(-R_{7}+C_{5})+\\ \;(R_{7}+R_{e}-C_{5}-R_{8})] \end{array}$$ (17)

$$\begin{array}{l} b_{32}=\frac{\partial F\left(z\right)}{\partial y}=z\left(1-z\right)\left[x\left(-R_{7}+C_{5}\right)+\left(R_{7}-C_{5}+D_{6}\right)\right] \end{array}$$ (18)

$$\begin{array}{l} b_{33}=\frac{\partial F(z)}{\partial z}=(1-2z)[xy(-R_{7}+C_{5})+x(R_{7}+R_{e}-C_{5}-R_{8})\\ \;+y(R_{7}-C_{5}+D_{6})-D_{6}] \end{array}$$ (19)

**Table 3:** Stability analysis of all equilibrium points.

**Equilibrium point**	**λ_1_, λ_2_, λ_3_**	**Symbol judgment**	**Stability**	**Condition**
*E*_1_ = (0, 0, 0)	*R*_1_ + *P*_*so*_ − *C*_1_ − *D*_2_ − *D*_3_ + *C*_2_ + *P*_1_ + *D*_1_, *R*_6_ + *R*_4_ − *C*_3_ + *C*_4_ + *D*_4_, − *D*_6_	(^*^, ^*^,-)	ESS	*A*
*E*_2_ = (0, 0, 1)	− R_*e*_ − *R*_3_ + *R*_1_ + *P*_*so*_ − *C*_1_ − *D*_2_ + *C*_2_ + *P*_1_ + *D*_1_, − *R*_5_ + *R*_6_ + *R*_4_ − *C*_3_ + *C*_4_ + *D*_4_, *D*_6_	(^*^, ^*^, +)	US	/
*E*_3_ = (0, 1, 0)	− *R*_*so*_ − *R*_2_ + *R*_1_ − *C*_1_ − *D*_3_ + *C*_2_ + *P*_1_ + *D*_1_, − (*R*_6_ + *R*_4_ − *C*_3_ + *C*_4_ + *D*_4_), − *R*_7_ − *C*_5_	(^*^, ^*^, ^*^)	ESS	*B*
*E*_4_ = (1, 0, 0)	− (*R*_1_ + *P*_*so*_ − *C*_1_ − *D*_2_ − *D*_3_ + *C*_2_ + *P*_1_ + *D*_1_), − *R*_*so*_ + *P*_*so*_ + *R*_4_ − *C*_3_ + *C*_4_ + *D*_4_, − *R*_7_ + *R*_*e*_ − *C*_5_ − *R*_8_ − *D*_6_,	(^*^, ^*^, ^*^)	ESS	*C*
*E*_5_ = (0, 1, 1)	− *R*_*e*_ − *R*_3_ − *R*_*so*_ − *R*_2_ + *R*_1_ − *C*_1_ + *C*_2_ + *P*_1_ + *D*_1_, − (− *R*_5_ + *R*_6_ + *R*_4_ − *C*_3_ + *C*_4_ + *D*_4_), (*R*_7_ − *C*_5_),	(^*^, ^*^, ^*^)	ESS	*D*
*E*_6_ = (1, 1, 0)	− (− *R*_*so*_ − *R*_2_ + *R*_1_ − *C*_1_ − *D*_3_ + *C*_2_ + *P*_1_ + *D*_1_), − (*R*_*so*_ + *P*_*so*_ + *R*_4_ − *C*_3_ + *C*_4_ + *D*_4_), *R*_*e*_ − *R*_8_ + *R*_7_ − *C*_5_	(^*^, ^*^, ^*^)	ESS	*E*
*E*_7_ = (1, 0, 1)	− (*P*_*so*_ − *R*_*e*_ − *R*_3_ + *R*_1_ − *C*_1_ − *D*_2_ + *C*_2_ + *P*_1_ + *D*_1_), *R*_*so*_ + *P*_*so*_ − *R*_5_ + *R*_4_ − *C*_3_ + *C*_4_ + *D*_4_, − (*R*_7_ + *R*_*e*_ − *C*_5_ − *R*_8_) + *D*_6_	(^*^, ^*^, ^*^)	ESS	*F*
*E*_8_ = (1, 1, 1)	*R*_*e*_ + *R*_3_ + *R*_*so*_ + *R*_2_ − *R*_1_ + *C*_1_ − *C*_2_ − *P*_1_ − *D*_1_, − (*R*_*so*_ + *P*_*so*_ − *R*_5_ + *R*_4_ − *C*_3_ + *C*_4_ + *D*_4_), − (*R*_7_ + *R*_*e*_ − *R*_8_ − *C*_5_)	(^*^, ^*^, ^*^)	ESS	*G*

^*^Condition *A*: *R*_1_ + *P*_*so*_ − *C*_1_ − *D*_2_ − *D*_3_ + *C*_2_ + *P*_1_ + *D*_1_ < 0, *R*_6_ + *R*_4_ + *C*_4_ + *D*_4_ − *C*_3_ < 0.

Condition *B*: − *R*_*so*_ − *R*_2_ + *R*_1_ − *C*_1_ − *D*_3_ + *C*_2_ + *P*_1_ + *D*_1_ < 0, *C*_3_ − *R*_6_ − *R*_4_ − *C*_4_ − *D*_4_ < 0, *R*_7_ − *C*_5_ < 0.

Condition *C*: $\begin{array}{c} -R_{1}-P_{so}+C_{1}+D_{2}+D_{3}-C_{2}-P_{1}-D_{1}<0,R_{\text{so}}+P_{\text{so}}+R_{4}+C_{4} \end{array}$$+D_{4}-C_{3}<0,R_{7}+R_{\text{e}}-C_{5}-R_{8}-D_{6}<0.$

Condition *D*: − *R*_e_ − *R*_3_ − *R*_so_ − *R*_2_ + *R*_1_ − *C*_1_ + *C*_2_ + *P*_1_ + *D*_1_ < 0, − *R*_5_ − *R*_6_ − *R*_4_ + *C*_3_ − *C*_4_ − *D*_4_ < 0, − *R*_7_ + *C*_5_ < 0.

Condition *E*: $R_{so}+R_{2}-R_{1}+C_{1}+D_{3}-C_{2}-P_{1}-D_{1}<0,-R_{\text{so}}-P_{\text{so}}-R_{4}+C_{3}-C_{4}-D_{4}<0,R_{\text{e}}-R_{8}+R_{7}-C_{5}<0.$

Condition *F*: $-\left(P_{\text{so}}-R_{\text{e}}-R_{3}+R_{1}-C_{1}-D_{2}+C_{2}+P_{1}+D_{1}\right)<0,R_{\text{so}}+P_{\text{so}}-R_{5}$$+R_{4}-C_{3}+C_{4}+D_{4}<0,-\left(R_{7}+R_{\text{e}}-C_{5}-R_{8}\right)+D_{6}<0.$

Condition *G*: $R_{\text{e}}+R_{3}+R_{\text{so}}+R_{2}-R_{1}+C_{1}-C_{2}-P_{1}-D_{1}<0,-$$\left(R_{\text{so}}+P_{\text{so}}-R_{5}+R_{4}-C_{3}+C_{4}+D_{4}\right)<0,-(R_{7}+R_{e}-R_{8}-C_{5})<0.$

### 3.2 Equilibrium analysis

**Proposition 1**. Under the condition *C*_1_ + *D*_2_ + *D*_3_ − (*R*_1_ + *P*_*so*_) > *C*_2_ + *P*_1_ + *D*_1_, *C*_3_ − (*R*_6_ + *R*_4_) > *C*_4_ + *D*_4_ and satisfaction of condition A, the evolutionary stable strategy of the game is *E*_1_ = (0, 0, 0).

The evolutionary result is that local governments implement negative support, social organizations choose non-effort strategies, and the older adults do not actively participate. In this equilibrium state, the implementation costs associated with active governmental support exceed those of inactive support measures, while social organizations face lower costs under non-effort scenarios. Consequently, local governments adopt inactive support policies, social organizations choose non effort strategies, and older adults participants demonstrate passive engagement. As the benefits derived from mutual older adults support systems decrease over time, participants progressively lose confidence and interest in these initiatives, leading to a decrease in participation rates. Consequently, this reinforcing cycle results in the older adults passive engage the mutual support.

**Proposition 2**. When *R*_*so*_ + *C*_1_ + *D*_3_ − *R*_1_ > *C*_2_ + *P*_1_ + *D*_1_ − *R*_2_, *C*_3_ − *R*_6_ − *R*_4_ < *C*_4_ + *D*_4_, *C*_5_ > *R*_7_ are satisfied, and the condition B is met, the evolutionary stable strategy of the game is *E*_3_ = (0, 1, 0).

The evolutionary result is that local governments implement negative support, social organizations choose effort strategies, and the older adults do not actively participate. In this case, local governments choose negative support because of the lower cost of it. For social organizations, their decision to exert effort emerges when the differential between effort cost *C*_3_ and aggregate benefits *R*_6_ + *R*_4_ falls below the combined value of non-effort cost C_4_ and potential losses *D*_4_. In this case, social organizations choose to participate in the effort strategy. Possible reasons are: With the aggravation of the problem of rural old-age care, local governments consider whether they need to vigorously support mutual-aid old-age care projects. As time evolves, local governments find that the cost of choosing positive support is higher than that of negative support. Under the pressure of finance, they choose negative support. Concurrently, social organizations maintain effort strategies due to favorable benefit-cost ratios. Under the negative support of local governments, the absence of robust governmental support and advocacy fails to transform traditional older adults care perceptions among the older adults. Moreover, the limited material and policy support inherent in negative government engagement compromises the long-term sustainability of mutual support systems, despite social organizations “active participation. This deterioration progressively erodes older adults participants' confidence and enthusiasm, so the older adults choose inactive participation.

**Proposition 3**. When *C*_1_ + *D*_3_ + *D*_2_ − (*R*_1_ + *P*_*so*_) < *C*_2_ + *P*_1_ + *D*_1_, *C*_3_ − (*R*_*so*_ + *R*_4_) > *C*_4_ + *D*_4_ + *P*_*so*_, *R*_7_ + *R*_*e*_ − *C*_5_ < *R*_8_ + *D*_6_ are satisfied, meet the condition C, the evolutionary stable strategy of the game is *E*_4_ = (1, 0, 0). In this case, local governments adopt active support strategies because the costs associated with inactive support exceed those of active support. Conversely, social organizations demonstrate reduced effort as their operational costs under active engagement surpass those of non-effort scenarios. For the older adults, the older adults strategic choice of inactive participation results from a rational evaluation of ‘free-rider' benefits, where the advantages of non-participation outweigh those of active engagement. In order to actively respond to the challenge of population aging, the government considers whether to actively support rural mutual pension in order to solve the people” s livelihood problems. In the case of measuring costs and benefits, it is found that the loss of negative support is greater than the cost and benefit of positive support, so local governments choose positive support. The benefits of social organization efforts are difficult to cover costs or the expected rate of return is not high, so social organizations choose not to work hard. For the older adults, the immediate benefits of free-riding behavior prove more attractive than those associated with active participation. Despite governmental resource allocation through active support measures, the reduced effort from social organizations results in suboptimal resource utilization and declining service quality. This deterioration in service delivery progressively erodes trust and enthusiasm among the older adults population, ultimately reinforcing their preference for inactive participation.

**Proposition 4** When *C*_1_ + *R*_*e*_ + *R*_*so*_ − *R*_1_ > *C*_2_ + *P*_1_ + *D*_1_ − (*R*_2_ + *R*_3_), (*R*_4_ + *R*_6_) − *C*_3_ > *R*_5_ − (*C*_4_ + *D*_4_), *C*_5_ < *R*_7_ are satisfied, meet the condition D, the evolutionary stable strategy of the game is *E*_5_ = (0, 1, 1).

The evolutionary result is that local governments implement inactive support, social organizations choose effort strategies, and the older adults participate actively. The cost-benefit analysis reveals that local governments' expenditure on active support measures significantly exceeds that of inactive support initiatives. Furthermore, social organizations demonstrate greater aggregate benefits through their effort compared to non-effort approaches. Given fiscal considerations and budgetary constraints, local governments strategically opt for inactive support mechanisms when the differential between active support's costs and benefits surpass that of inactive support measures. From the perspective of economic benefits, the net income of social organizations is higher than that of non-efforts. It is beneficial to choose to participate in mutual assistance and self-development, so they choose to effort. For the older adults, although the government chooses inactive support, social organizations try to participate in the provision of older adults care services. Out of their older adults care needs and their own economic considerations, the benefits of their active participation are greater than the costs, so the older adults choose to actively participate.

**Proposition 5** When *R*_1_ − (*C*_1_ + *D*_3_ + *R*_*so*_) > *R*_2_ − (*C*_2_ + *P*_1_ + *D*_1_), *C*_3_ − (*R*_*so*_ + *R*_4_) < *P*_*so*_ + *C*_4_ + *D*_4_, *R*_*e*_ + *R*_7_ − *C*_5_ < *R*_8_ are satisfied, meet the condition *E*, the evolutionary stable strategy of the game is *E*_6_ = (1, 1, 0).

This equilibrium emerges because the net benefit of local government's active support exceeds that of inactive support, while social organizations' effort costs are outweighed by their benefits. Additionally, the “free-riding” utility for older adults inactive participants surpasses the benefits of active participation. Which means that under the challenge of population aging, the positive externalities generated by government's active support demonstrate higher utility compared to inactive support approaches. At the same time, when local governments provide active support, they create an enabling environment for social organizations, effectively reducing these organizations' operational costs, even without active participation in mutual older adults care, the rural older adults can still benefit from the infrastructure and services provided by local governments. Therefore, the evolutionary result is that local governments implement active support, social organizations choose effort strategies, and the older adults inactive participate.

**Proposition 6** When *P*_*so*_ + *R*_1_ − (*R*_*e*_ + *C*_1_ + *D*_2_) > *R*_3_ − (*C*_2_ + *P*_1_ + *D*_1_), (*R*_*so*_ + *R*_4_) − *C*_3_ < *R*_5_ − (*C*_4_ + *D*_4_ + *P*_*so*_), *R*_7_ + *R*_*e*_ − (*C*_5_ + *D*_6_) > *R*_8_ are satisfied, meet the condition F, the evolutionary stable strategy of the game is *E*_7_ = (1, 0, 1).

In this equilibrium scenario, local governments opt for active support strategies when the utility derived from active support exceeds that of inactive support. However, the presence of robust government support may induce dependency behavior among social organizations, leading them to perceive reduced necessity for resource and effort investment, response manifests in social organizations selecting non-effort strategies. The rural older adults care context presents both immediate and pressing challenges. The intersection of limited economic resources and heightened demand for older adults care services creates a distinct operational environment. Under these conditions, government active support provides essential infrastructure and resources for older adults care services. Notably, the utility derived by the older adults from active participation exceeds the potential gains from free-riding behavior. Consequently, despite the reduced effort from social organizations, the older adults population demonstrates a preference for active participation in mutual support systems.

**Proposition 7** When *R*_*e*_ + *R*_*so*_ + *C*_1_ − *R*_1_ < *C*_2_ + *P*_1_ + *D*_1_ − (*R*_2_ + *R*_3_), *R*_*so*_ + *R*_4_ − *C*_3_ > *R*_5_ − (*P*_*so*_ + *C*_4_ + *D*_4_), *R*_7_ + *R*_*e*_ − *C*_5_ > *R*_8_ are satisfied, meet the condition F, the evolutionary stable strategy of the game is *E*_8_ = (1, 1, 1).

Under these conditions, local governments adopt active support strategies when the negative consequences of passive support exceed the costs associated with active support implementation. This strategic choice by local governments creates a cascading effect through the system. The government's active support manifests through policy incentives, resource allocation, and enhanced social benefits, fundamentally altering the utility calculations for other stakeholders. For social organizations, this governmental support creates an environment where the aggregate benefits of participation exceed the marginal benefits of non-effort strategies. Consequently, they rationally select effort strategies, allocating resources and energy toward the older adults care initiatives. The coordinated efforts of local governments and social organizations generate positive externalities that influence older adults participation decisions. The rural older adults population experiences enhanced trust and security within this support framework, perceiving tangible opportunities to address their care needs and improve their quality of life through active participation. The perception of the older adults shifts toward to active engagement.

## 4 Simulation analysis

To analyze the impact of various elements in the replication dynamic system on the evolutionary game process and outcomes, numerical simulations were conducted using MATLAB 2018b. These simulations also validate the evolutionary stability analysis. The simulations focused on analyzing the evolutionary trajectories of various stakeholders in the mutual older adults care. Based on actual situation, we first examined the potential equilibrium points, specifically: the government's supportive policies, social organizations “involvement levels, and older adults participants” engagement rates. According to Proposition 4, the conditions for achieving a stable equilibrium point (0, 1, 1) in the endogenous mutual assistance pension system are as follows: ( − *R*_*e*_ − *R*_3_ − *R*_*so*_ − *R*_2_ + *R*_1_ − *C*_1_ + *C*_2_ + *P*_1_ + *D*_1_), − (− *R*_5_ + *R*_6_ + *R*_4_ − *C*_3_ + *C*_4_ + *D*_4_), − (*R*_7_ − *C*_5_). The variable data is artificially simulated and assigned to analyze the influence trend of different parameter changes on the behavior of participants. The corresponding parameter assignments are shown in Table 4.

Table 4Parameter assignment.

**Table:** 

**Parameter assignment**	***C*_1_**	***C*_2_**	***C*_3_**	***C*_4_**	***C*_5_**	***R*_1_**	***R*_2_**	***R*_3_**	***R*_4_**	***R*_5_**
	10	5	15	4	4	15	5	3	14	5

**Table:** 

**Parameter assignment**	***R*_7_**	***R*_8_**	***R*_*e*_**	***R*_*so*_**	***P*_1_**	***P*_*so*_**	***D*_1_**	***D*_4_**	***x***	***y***	***z***
	13	5	4	6	4	5	3	5	0.5	0.5	0.5

### 4.1 The influence of initial willingness on the behavior of participants

#### 4.1.1 Evolution path of initial intention

**Figure 2:**
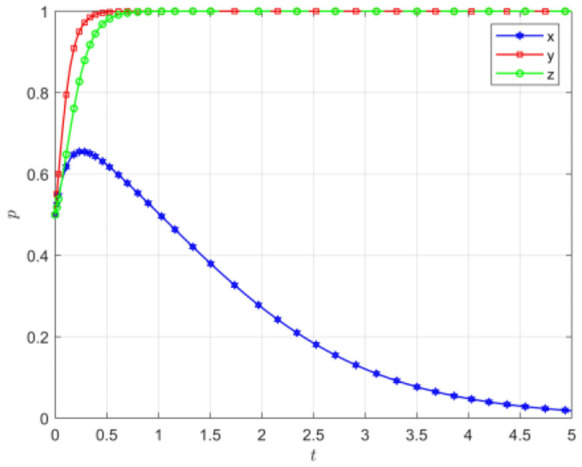
Initial intention evolution path diagram.

Assuming that the initial willingness of the three parties in the game is *x* = 0.5, *y* = 0.5, *z* = 0.5, other parameters are set as shown in Table 4, and the initial evolution simulation results are shown in Figure 2.

Figure 2 demonstrates that during the construction of the rural mutual aid pension model, when the initial willingness of all three parties is set at 0.5, both social organizations and rural older adults populations demonstrate a tendency toward positive engagement. Initially, local governments actively encourage and support social organizations while promoting older adults participation in the mutual aid pension system. In this phase, social organizations active engage, while the older adults demonstrate consistent active participation in the program. As these two stakeholder groups progressively evolve toward active participation in the endogenous mutual older adults care model and achieve equilibrium, the government's role undergoes a significant transformation. The government gradually transitions from direct involvement in the mutual aid pension project to a supervisory position. This shift creates expanded opportunities for social organizations and the older adults to exercise self-management. they can autonomously engage in the mutual older adults care.

#### 4.1.2 The influence of the initial willingness changes of the three parties on the evolution of the system

**Figure 3:**
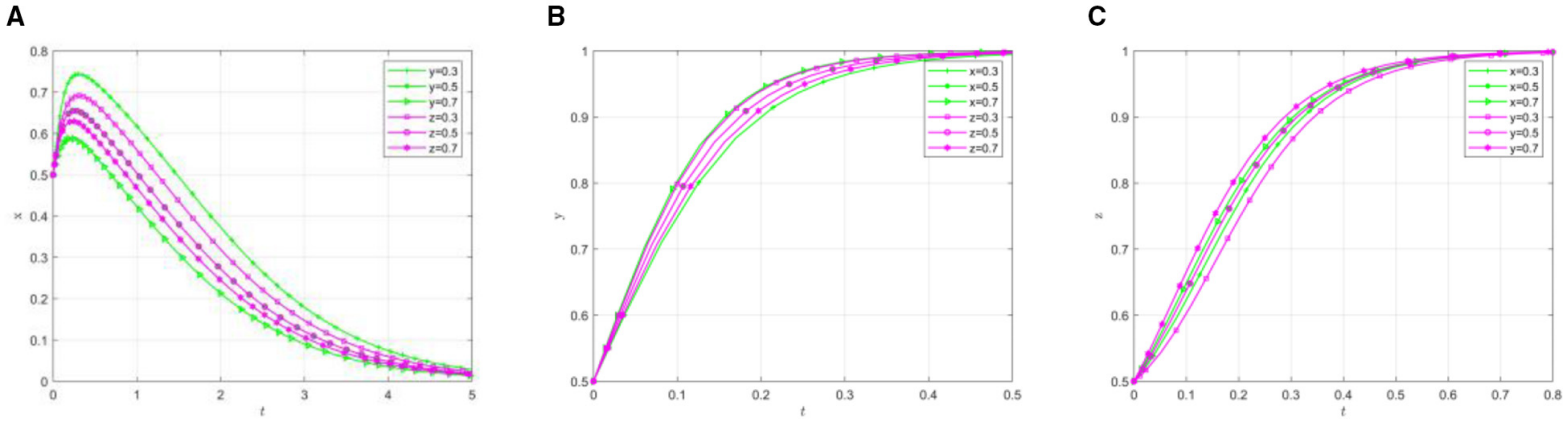
Effect of parameter variation on the evolutionary results under different initial conditions. **(A)** The effect of local government by changing y/z. **(B)** The effect of social organization by changing x/z. **(C)** The effect of the older adults by changing x/y.

Other parameters remain unchanged, change the initial participation willingness of the three parties, and analyze its impact on the selection strategy of the participants. The evolution results are shown in Figure 3.

As shown in Figure 3, the increase in the initial willingness of social organizations and older adults participants leads to a decline in local governments' active support, subsequently accelerating their transition toward negative support. Specifically, heightened governmental participation willingness reduces the time required for social organizations to reach equilibrium, while increased older adults participation willingness extends the time to reach equilibrium. As the willingness of both local governments and social organizations increases, the rate of active participation among the older adults accelerates accordingly. The simulation results demonstrate that while high initial willingness promotes active participation from social organizations and the older adults, it simultaneously expedites local governments” shift toward inactive support. This phenomenon may be attributed to the fact that high initial willingness facilitates active participation of social organizations and the older adults, enabling mutual-aid pension projects to gradually develop self-management capabilities. Consequently, local governments tend to transition from direct support to a regulatory role, progressively adjusting their position within the mutual older adults care.

### 4.2 The influence of reward intensity on the behavior of participants

**Figure 4:**
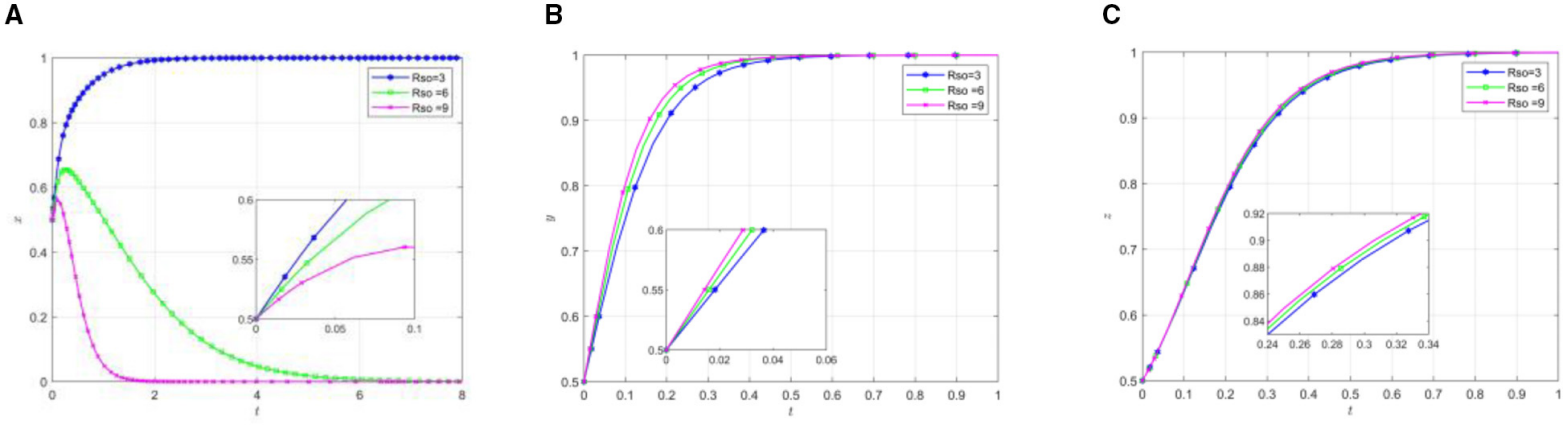
The impact of local governments on social organization awards. **(A)** The effect of local government by changing *R*_*so*_. **(B)** The effect of social organization by changing *R*_*so*_. **(C)** The effect of the older adults by changing *R*_*so*_.

**Figure 5:**
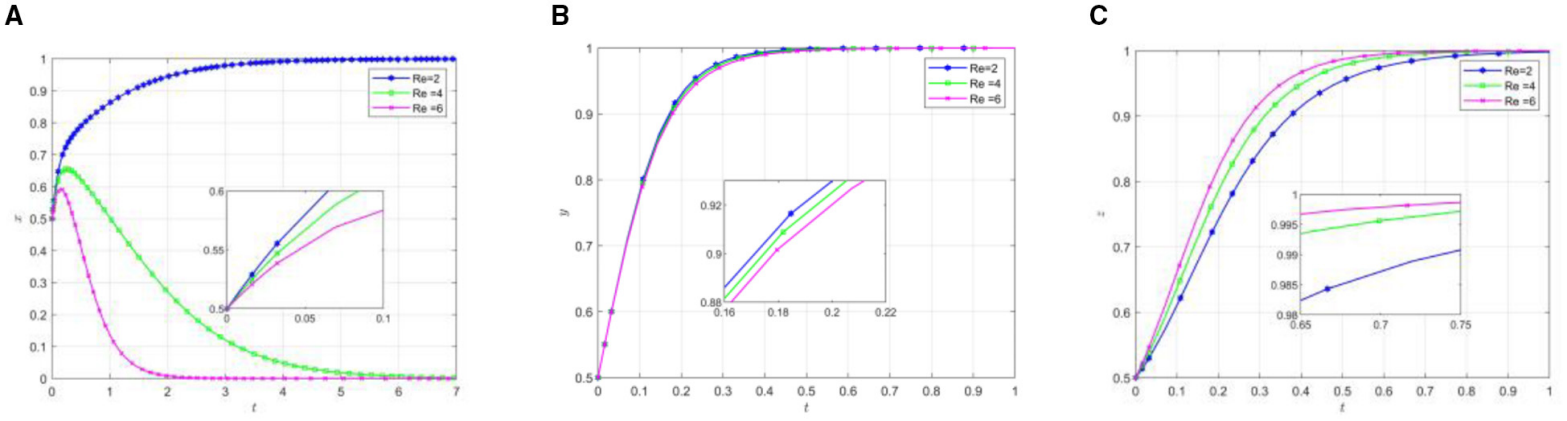
The impact of local governments on rural older adults rewards. **(A)** The effect of local government by changing *R*_*e*_. **(B)** The effect of social organization by changing *R*_*e*_. **(C)** The effect of the older adults by changing *R*_*e*_.

To investigate how local government incentives provided to social organizations and older adults rural residents influence the evolutionary path of rural endogenous mutual older adults care participants, various incentive parameters *R*_*so*_ and *R*_*e*_ were established for model simulation. Figures 4, 5 illustrate the evolutionary trajectories of participant behavior under different incentive conditions.

#### 4.2.1 Local government' awards to social organizations

As observed in Figure 4, the evolutionary results demonstrate that as local governments increase their incentives for social organizations, there is an accelerated evolution in the participation efforts of social organizations. Concurrently, local governments exhibit an evolutionary trend that transitions from active to inactive support. This phenomenon can be attributed to several factors. Firstly, the enhancement of incentive intensity directly increases the rate of return for social organizations, gradually establishing “active participation” as their dominant strategy. Second, examining the strategic choices of local governments reveals a strong initial willingness to provide support, which aligns with the policy orientation of promoting rural mutual older adults care. However, as time progresses, social organizations and the older adults have gradually developed a self-managed, endogenous mutual assistance pension model. At this stage, the cost associated with adopting a negative support strategy becomes lower than that of maintaining positive support, prompting local governments to shift their strategic choice from active to inactive support. The initial positive support from local governments serves as a catalyst for establishing mutual assistance systems. Once these systems achieve a certain level of self-sustainability, local governments appear to recalibrate their involvement, potentially optimizing resource allocation while maintaining system stability.

#### 4.2.2 Local government' awards to the rural older adults

Figure 5 reveals the dynamic evolutionary patterns in response to increased local government incentives for older adults participation. The results indicate that higher rewards for the older adults correlate with an accelerated rate of active participation among this demographic. While local governments initially demonstrate positive support, they eventually transition toward negative support at an increasing rate. Notably, as government rewards for the older adults increase, social organizations exhibit a declining probability of participatory effort, accompanied by a decelerated evolution rate. This phenomenon can be attributed to several underlying mechanisms. Initially, local governments adopt an active support strategy to promote older adults participation in mutual older adults care, aligning with policy objectives for rural older adults care development. As the system matures, both the older adults and social organizations become increasingly engaged, contributing to the establishment of a more sophisticated endogenous mutual support framework. During this maturation phase, local governments strategically transition from direct support to a supervisory role, gradually withdrawing from direct involvement in mutual support operations. This transition grants greater autonomy to both social organizations and the older adults participants. The increased rewards for the older adults during this phase correlate with deeper and more extensive participation patterns. However, the government's withdrawal from its supportive role in the later stages introduces additional operational challenges for social organizations, manifesting in increased costs and management complexity. These factors collectively contribute to the observed deceleration in social organizations' progression toward equilibrium.

Comparative analysis of Figures 4, 5 reveals distinct patterns in the effectiveness of local government incentive mechanisms. The results demonstrate that incentives directed toward social organizations generate positive behavioral responses from both social organizations and the older adults population. In contrast, incentives targeted specifically at the older adults population, while successful in promoting older adults participation, exhibit an inverse relationship with social organization engagement.

The empirical evidence suggests that incentives for social organizations produce a more comprehensive and synergistic effect on the mutual pension system. This finding holds particular significance for policy implementation, as it indicates that channeling incentives through social organizations may yield more efficient outcomes in promoting rural mutual pension programs.

### 4.3 The influence of punishment on the behavior of participants

#### 4.3.1 Punishment of local governments by higher authorities

**Figure 6:**
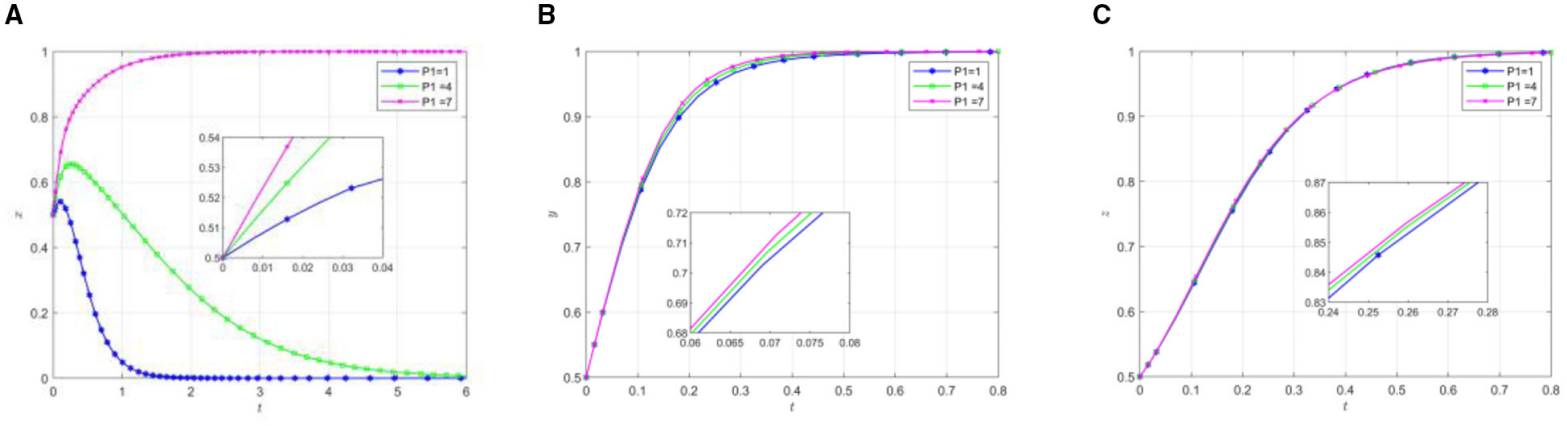
The influence of superior government on local government punishment. **(A)** The effect of local government by changing P_1_. **(B)** The effect of social organization by changing P1. **(C)** The effect of the older adults by changing P_1_.

To analyze the impact of different punishment intensities imposed by superior governments on local governments and other participants, simulation experiments were conducted with varying punishment parameters *P*_1_. Other parameters were kept constant, as specified in Table 4. The simulation results demonstrating how different punishment levels affect the behavioral patterns of various stakeholders are illustrated in Figure 6.

Analysis of Figure 6 reveals that within the evolutionary system, increasing punishment intensity from the superior government shifts the behavioral strategy equilibrium points of local governments, social organizations, and older adults participants from (0,1,1) to (1,1,1). This transition can be attributed to the enhanced punishment mechanism for local governments' inactive support, which effectively incentivizes them to adopt positive support strategies. The subsequent positive policy environment created by local governments' supportive stance facilitates more rapid strategy adoption by social organizations. Furthermore, the synergistic effect of local governments “positive support and social organizations” effort engagement leads to substantial improvements in the older adults quality of life. This improvement in tangible benefits consequently increases the older adults population's perceived value of participation, thereby promoting their active engagement in the system.

#### 4.3.2 Punishment of social organizations by local governments

**Figure 7:**
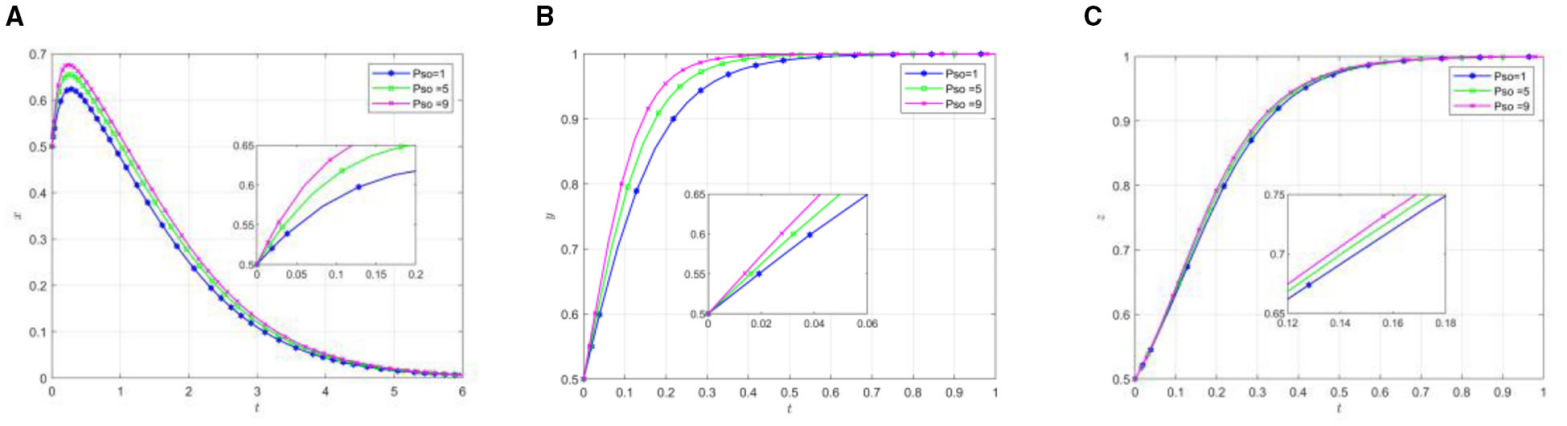
The influence of local governments on the punishment of social organizations. **(A)** The effect of local government by changing Pso. **(B)** The effect of social organization by changing *P*_*so*_. **(C)** The effect of the older adults by changing *P*_*so*_.

To thoroughly analyze the impact of varying punishment intensities imposed by local governments on the behavior of social organizations and other participants, this study conducted model simulations with different punishment strengths *P*_*so*_. Other parameters were kept constant, as shown in Table 4. Figure 7 illustrates the evolutionary game process of participating subjects under different punishment intensities.

Analysis of Figure 7 reveals several key dynamics in the evolutionary system. As the punishment intensity on social organizations increases, two primary effects are observed. First, the government's positive support rate accelerates during the initial phase. Second, the evolution toward negative support decelerates. Additionally, social organizations demonstrate faster adoption of participatory efforts, while the older adults show a quicker but relatively smaller increase in active participation. This phenomenon can be attributed to punishment serving as an effective regulatory mechanism for local governments to influence social organizations' behavior. The accelerated shift toward active government support appears to be motivated by the objective of promoting social organizations' engagement in mutual older adults care support initiatives. Under the pressure of potential government sanctions, social organizations are compelled to adopt effort-based strategies. Consequently, the older adults inclination toward active participation can be understood as a response to the enhanced benefits derived from increased organizational effort.

The comparative analysis of simulation results from Figures 6, 7 demonstrates patterns in behavioral evolution. Higher-level government sanctions emerge as a significant driver in modifying local government behavioral strategies, while simultaneously accelerating the adoption of positive behaviors by both social organizations and the older adults. In contrast, variations in local government punitive measures toward social organizations exhibit minimal impact on participants' evolutionary speed and do not alter the equilibrium outcomes. These findings suggest that enhancing higher-level government punishment mechanisms serves as a more effective approach in guiding the three participating entities toward a positive equilibrium state at an accelerated rate.

### 4.4 The impact of reputation loss on the behavior of participants

#### 4.4.1 The impact of local government reputation loss on system evolution

**Figure 8:**
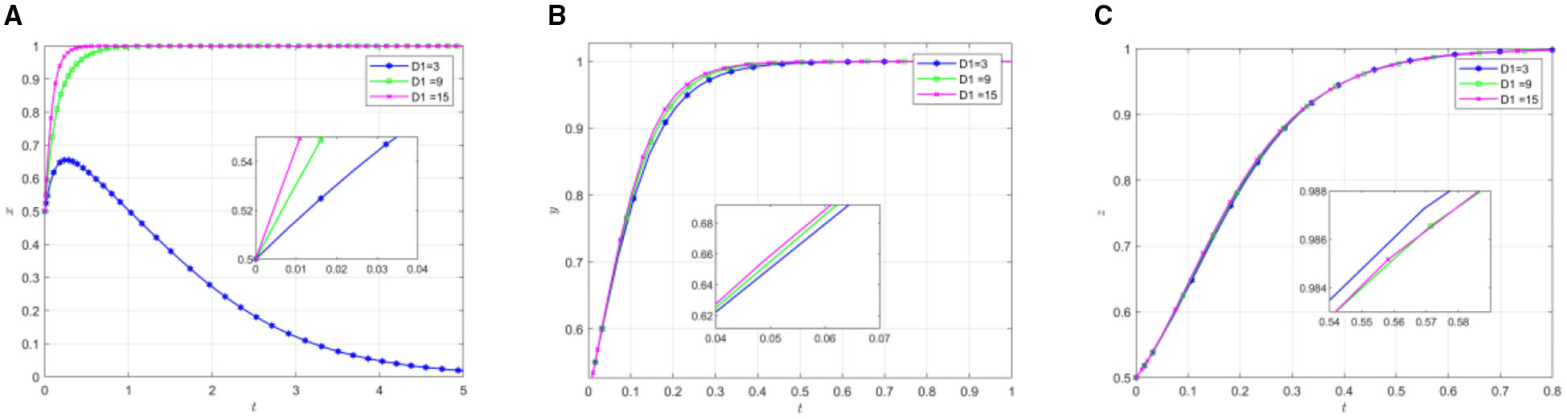
The impact of local government reputation loss. **(A)** The effect of local government by changing D1. **(B)** The effect of social organization by changing D_1_. **(C)** The effect of the older adults by changing D_1_.

In order to observe the influence of local government reputation loss on the strategic choice of rural endogenous mutual pension participants, the other parameters are shown in Table 4. By setting different reputation loss degree*D*_1_, the process of participating in the evolution of the main strategy is shown in Figure 8.

Figure 8 illustrates that increasing local government reputation loss shifts the system's equilibrium point from (0,1,1) to (1,1,1). As reputation loss intensifies, two distinct temporal patterns emerge: social organizations demonstrate accelerated evolution, while the older adults exhibits initial rapid acceleration in active participation followed by a decelerated rate with minimal fluctuation.

These behavioral dynamics can be attributed to several factors. When government reputation loss reaches a critical threshold, local governments tend to adopt supportive strategies to mitigate further reputational damage. Under conditions of compromised government reputation, public scrutiny of social organizations intensifies. This heightened attention provides opportunities for social organizations to improve their societal position and reputation through positive engagement. As a result, they can enhance their ability to attract donations, volunteers, and strategic partnerships while expanding their influence.

The older adults response pattern reflects a complex interplay between long-term trust and temporal dynamics. Initially, their deep-rooted trust in governmental institutions, built on accumulated experience, renders them relatively resilient to short-term reputational fluctuations, maintaining their active participation. However, as time progresses, a gradual erosion of confidence in governmental institutions appears to contribute to a decline in active participation rates.

#### 4.4.2 The impact of social organization reputation loss on system evolution

**Figure 9:**
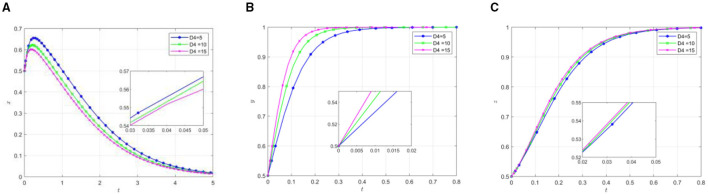
The impact of social organization reputation loss. **(A)** The effect of local government by changing D_4_. **(B)** The effect of social organization by changing D4. **(C)** The effect of the older adults by changing D_4_.

Analysis of Figure 9 reveals distinctive behavioral patterns in response to increasing reputational losses of social organizations. Three primary trends emerge: a deceleration in local governments “evolution toward active support, an acceleration in social organizations” effort-based evolution, and an increased rate of active participation among the older adults population. This behavioral dynamic can be attributed to multiple interacting factors. When social organizations experience reputational damage due to insufficient effort, local governments tend to reduce their support based on three key considerations: protection of public interest, financial efficiency, and preservation of governmental image and accountability. Under these circumstances, social organizations are compelled to pursue effort-based strategies as the primary means of rehabilitating their social standing and mitigating reputational damage through positive social evaluation. The older adults response demonstrates a notably pragmatic adaptation. Despite the compromised reputation of social organizations, the continued availability of services and programs, coupled with “free-rider” benefits and fundamental eldercare needs, renders the older adults population relatively resilient to short-term reputational fluctuations, maintaining their active participation patterns.

Comparative analysis of simulation results from Figures 8, 9 reveals differential impacts of reputational losses on system dynamics. Local government reputational losses demonstrate substantial influence on both equilibrium outcomes and evolutionary velocities, facilitating accelerated behavioral adjustments among local governments while catalyzing increased participation from both social organizations and the older adults. In contrast, while social organizations' reputational losses induce some degree of self-correcting behavioral modifications, their impact manifests as a dual effect: generating internal behavioral corrections within social organizations while simultaneously impeding local governments' progression toward active support strategies.

## 5 Conclusion and management enlightenment

### 5.1 Conclusions

This study investigates the development of a rural endogenous mutual older adults care model through a tripartite evolutionary game theoretical approach. The research examines the strategic interactions between local governments, social organizations, and rural older adults populations under bounded rationality conditions. Using software Matlab 2018b for numerical simulation and sensitivity analysis. This study yields several significant findings as follows:

Firstly, the equilibrium state of (active support, effort, active participation) emerges as the optimal configuration for implementing the endogenous mutual-aid pension model. This equilibrium demonstrates a sequential dynamic where initial governmental support encourages participation from both social organizations and the older adults. As these latter two groups establish stable cooperative patterns, government support gradually diminishes, highlighting the government's crucial role in catalyzing but not perpetually maintaining the system.

Secondly, the evolution and equilibrium outcomes of participant behavior are primarily influenced by four key factors: initial participation willingness, incentive intensity, punitive measures, and reputational consequences. The analysis reveals that higher initial participation willingness among social organizations and the older adults accelerates governmental equilibrium achievement, though following a consistent pattern of initial positive support transitioning to active support. Interestingly, increased initial older adults participation correlates with slower equilibrium attainment by social organizations.

Thirdly, enhanced incentives for social organizations and older adults participants expedite the government's transition to active support strategies. However, the relationship is not uniform: increased older adults-focused incentives decelerate social organizational evolution, while enhanced organizational incentives accelerate both organizational and older adults equilibrium achievement, ultimately facilitating faster model implementation.

The fourth conclusion, the increase in incentives for social organizations and the older adults will prompt local governments to choose negative support strategies faster, and the increase in incentives for the older adults will slow down the evolution of social organizations. The increase in incentives for social organizations will promote social organizations and the older adults to reach a balanced state faster, and accelerate the generation of endogenous mutual support pension models.

Furthermore, intensified higher-level governmental oversight and sanctions promote more rapid adoption of active support strategies by local governments and accelerate overall tripartite equilibrium achievement. The study also finds that stronger local government sanctions on social organizations decelerate the transition to negative governmental support while accelerating organizational and older adults equilibrium achievement.

Finally, the reputation loss of local government can accelerate the adjustment of local government behavior and promote social organizations to choose positive behavior more quickly; the reputation loss of social organizations has a corrective effect on themselves, but hinders the positive behavior of local governments.

### 5.2 Management enlightenment

#### 5.2.1 Establishing a differentiated reward-punishment mechanism to catalyze endogenous motivation

Our simulation results reveal that the effects of reward intensity and punishment severity vary significantly across different stakeholders, necessitating a differentiated institutional design rather than uniform policy instruments. From a vertical governance perspective, higher-level governments should enhance their supervisory role. This can be achieved by implementing appropriate penalties for local governments' negative behaviors, thereby encouraging active support for older adults mutual support initiatives. At the horizontal governance level, local governments must act as facilitators. They should enhance penalty mechanisms to increase the opportunity costs of adverse behaviors by social organizations. This compels these organizations to share external losses with both the government and the older adults. Furthermore, it is essential to establish a comprehensive support and incentive system for social organizations. This system should provide multifaceted assistance in terms of policy implementation, resource allocation, project development, and technical support. While tax relief and operational subsidies can encourage social organization participation, the intensity of these incentives must be carefully calibrated. The intensity should avoid imposing an excessive financial burden, which could compromise the sustainable development of older adults mutual support programs. Additionally, implementing appropriate incentive mechanisms for older adults participants can foster a virtuous cycle where social organizations' involvement leads to enhanced benefits for the older adults.

#### 5.2.2 Enhancing social organizations' capacity to mitigate the “willingness paradox”

The operational efficacy of the rural mutual older adults care model fundamentally depends on the capabilities of social organizations. However, our model reveals a counterintuitive finding: high willingness from social organizations may inadvertently accelerate local governments' tendency toward passive support, as proactive organizational engagement alleviates perceived governmental pressure. To address this paradox while strengthening organizational capacity, social organizations should pursue a dual strategy. First, they must enhance professional capacity through strategic recruitment of qualified professionals and certified social workers with specialized expertise in older adults care services, alongside integration of advanced information technology systems to improve service delivery. Second, organizations should maintain transparent communication regarding service gaps and resource constraints, ensuring that governmental support obligations remain visible rather than obscured by organizational overreach.

The distinction between exogenous and endogenous factors carries critical implications for organizational sustainability. External subsidies and policy preferences constitute exogenous inputs that may fluctuate with governmental priorities. In contrast, professional expertise, institutional reputation, community trust relationships, and adaptive management capabilities represent endogenous organizational assets. Sustainable development requires systematically converting initial exogenous support into accumulated endogenous capital, thereby reducing long-term vulnerability to external policy shifts. Such systematic capacity building that balances competence enhancement with strategic positioning is essential for fostering sustainable development of the endogenous mutual older adults care model.

#### 5.2.3 Constructing a tripartite coordination mechanism based on evolutionary game equilibrium

Collaborative governance among multiple stakeholders constitutes the fundamental element of rural mutual older adults care systems. The sustainable development of rural endogenous mutual assistance and older adults care necessitates the coordinated participation of government entities, social organizations, rural older adults populations, and other stakeholders, facilitating shared responsibilities and benefits while accelerating the formation of a multi-governance framework. Local governments must adhere to the principle of “government-led, social autonomy” in this collaborative framework (25).

Specifically, local governments must therefore adopt a phased approach aligned with system evolution. In the initial phase, exogenous interventions establish baseline behavioral expectations and demonstrate credible commitment to mutual care initiatives. As the system evolves, policy emphasis should transition toward cultivating endogenous coordination mechanisms—strengthening inter-stakeholder communication platforms, facilitating trust-building interactions, and enhancing the visibility of reputation effects that reward cooperative behavior through community recognition rather than material incentives.

In parallel, social organizations play a pivotal role in this exogenous-to-endogenous transition by serving as intermediaries that translate external policy signals into community-level relational dynamics. Through their roles in publicity, guidance, mobilization, and capacity building, organizations can transform externally mandated participation into internally motivated community engagement. Through these functions, they can effectively mobilize diverse resources, stimulate older adults participation, and inject sustainable momentum into rural mutual older adults care initiatives, ultimately enhancing their organizational credibility and brand reputation.

## Data Availability

The original contributions presented in the study are included in the article/supplementary material, further inquiries can be directed to the corresponding author.
